# An Investigation Into the Prognostic Significance of High Proteasome PSB7 Protein Expression in Colorectal Cancer

**DOI:** 10.3389/fmed.2020.00401

**Published:** 2020-08-07

**Authors:** Ju-Yoon Yoon, Julia Y. Wang, Michael H. A. Roehrl

**Affiliations:** ^1^Department of Laboratory Medicine and Pathobiology, University of Toronto, Toronto, ON, United States; ^2^Curandis, New York, NY, United States; ^3^Department of Pathology, Memorial Sloan Kettering Cancer Center, New York, NY, United States; ^4^Human Oncology and Pathogenesis Program, Memorial Sloan Kettering Cancer Center, New York, NY, United States

**Keywords:** PSB7, colorectal cancer, prognosis, biomarker, oncology, protein expression, proteasome

## Abstract

Using unbiased proteomics, we had previously discovered that the catalytic proteasome subunit β type 7 (PSB7) protein is frequently overexpressed in colorectal adenocarcinomas. In this paper, we validate this finding and derive a prognostic significance for PSB7 by examining an expanded, well-annotated clinical cohort of 318 colorectal cancer patients. We found PSB7 protein levels to be similarly increased in both advanced stage primary disease and metastatic lesions. We then examined the prognostic value of PSB7 protein expression. Elevated PSB7 protein as well as *PSMB7* mRNA levels showed associations with lower overall survival, particularly in female patients. The prognostic value of elevated PSB7 protein levels was highest for female patients who were older (>60 years of age at diagnosis) or who had received adjuvant chemotherapy. While high PSB7 did not retain its prognostic significance on multivariate analysis, we discuss the potential significance of PSB7 as a biomarker, considering its differential prognostic strength in different colorectal cancer patient groups and given its role as a subunit of the immunoproteasome for antigen presentation.

## Introduction

Colorectal cancer is the fourth most common cancer and the second most common cause of cancer-related deaths in the US (1). Previously, we had examined paired cancerous and normal clinical tissue specimens from patients with colorectal adenocarcinomas using heparin affinity fractionation enrichment (HAFE), which allowed for the enrichment of low-abundance proteins (2). Enriched proteins were identified by 2-D difference gel electrophoresis and subsequent tandem mass spectrometric (MS/MS) identification. Using this approach, we had discovered three differentially expressed proteins, namely proteasome subunit β type 7 (PSB7), peroxiredoxin-1 (PRDX1), and signal recognition particle 9 kDa protein (SRP9). PSB7 was found to be overexpressed in colorectal cancer samples by both immunoblotting and immunohistochemistry, and it was found to be overexpressed in the cytoplasm, as well as the nuclei, of the cancerous epithelial cells.

*PSB7* encodes the β7 subunit of the 20S proteolytic core of the 26S proteasome, and one of the catalytically active subunits (3). The eukaryotic 26S proteasome consists of the catalytic 20S proteasome and two regulatory 19S complexes, forming a large cylindrical complex. The 20S catalytic component is composed two outer rings composed of seven α subunits (α1-7) and two inner rings of seven β subunits (β1-7), including PSB7. Outside of the results from our proteomic screen, there are no reported associations between PSB7 and colorectal cancer. In breast cancer, higher PSB7 expression was associated with poorer survival, and silencing the gene resulted in greater sensitivity to doxorubicin *in vitro* (4). PSB7 is phosphorylated, and, in a small series examining several different cancer cell lines *in vitro*, the level of phosphorylated PSB7 was significantly lower in tumor cells than in non-tumoral cells (5).

In this study, we examined the prognostic significance of PSB7 protein expression in colorectal cancer, and we found high PSB7 levels to be associated with poorer overall survival in female patients, especially those who are older and those who received adjuvant chemotherapy. Possible mechanisms for this observation are discussed, namely the dynamic stoichiometric balance between proteasome and immunoproteasome.

## Materials and Methods

### Patient Samples and Tissue Microarrays

Our cohort consisted of 318 cases of colorectal cancer treated between 1994 and 2008 at the University Health Network (UHN, Toronto). This study was approved by UHN's Research Ethics Board (REB), and all experiments were carried out according to relevant institutional regulations and guidelines. The study used only fully de-identified retrospective material that had been collected as part of routine clinical care. Thus, the UHN REB had determined that informed consent was waived. Tissue microarrays (TMAs) were constructed as described previously (6). For each case, four to eight 0.6-mm and 1.5-mm cores were obtained from formalin-fixed and paraffin-embedded tissue donor blocks and transferred into TMA blocks using a manual tissue arrayer (Beecher Instruments). Hematoxylin and eosin (H&E) stained slides of these cases were reviewed for the presence of normal colonic mucosa, adenoma, and adenocarcinoma by an experienced gastrointestinal pathologist (MHR).

### Immunohistochemistry (IHC)

IHC was performed as described previously (7). Briefly, formalin-fixed and paraffin-embedded tissue core TMAs were used, and PSB7 protein was detected by PSB-specific antibodies (clone HN3, AbCam; dilution 1:200) and peroxidase-DAB (diaminobenzidine) chemistry using the NovoLink Polymer Detection System (Vision BioSystems) after microwave boiling in 5% (m/v) urea in Tris-buffered saline. The total weighted IHC score (IHC H-score) of a sample was calculated by multiplying the expression intensity of individual tumor areas (score, 0–3) by their relative contribution (0–100%) to total tumor area and adding these to yield a total weighted sum. The IHC H-scores thus have a theoretical range of 0–300. Tumor samples were categorized into high vs. low PSB7 protein expression levels based on whether the H-score for each sample was higher or lower than the median H-score for the 318 tumor case cohort.

### Statistics and Survival Analyses

Comparisons of the PSB7 H-scores among categories were performed by Kruskal-Wallis or Mann-Whitney tests. Overall and recurrence-free survival were examined using the Kaplan-Meier method, and survival curves were generated using the JMP 11.0 software (SAS). Log-rank tests were used to examine differences in survival. A Cox proportional hazards model was employed to examine survival in multivariate analyses.

### Transcriptional Analysis

The Cancer Genome Atlas (TCGA) data was accessed from cBioPortal (http://www.cbioportal.org/). Both the clinical and mRNA (RNA-Seq) datasets for colorectal cancer were retrieved. The TCGA cohort was dichotomized into high and low *PSMB7* mRNA transcriptional groups, using the median mRNA level as the cut-off.

## Results

### Associations Between PSB7 Levels and Colorectal Cancer Patient Characteristics

**Figure 1:**
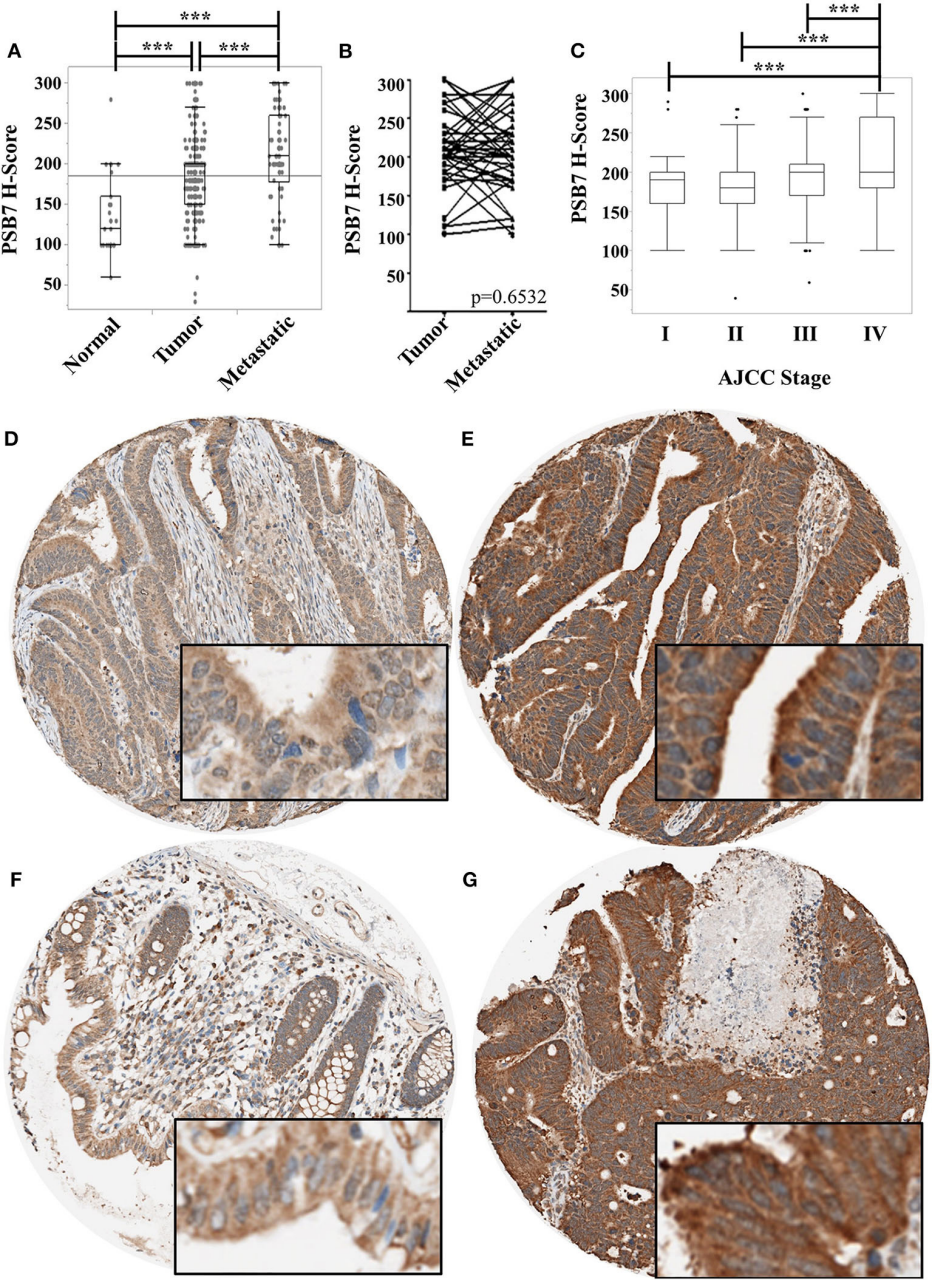
Comparison of PSB7 levels by immunohistochemistry. **(A)** Comparison of levels between normal colorectal surface epithelium, primary colorectal cancer lesions, and metastatic colorectal cancer lesions. **(B)** Comparison of PSB7 levels among the matched primary and metastatic cancer lesions. **(C)** Comparison of PSB7 levels among the different clinical AJCC stages in primary colorectal tumor samples. **(D)** Representative tumor cores with low H-score. **(E)** Representative tumor cores with high H-score. **(F)** Representative normal colon core staining. **(G)** Representative metastatic lesion core. ***p < 0.0001.

We had previously reported the levels of PSB7 to be higher in colorectal cancer samples by mass spectrometry, immunoblotting, and immunohistochemistry (2). We expanded our cohort using tissue microarrays (TMAs) to examine PSB7 levels by immunohistochemistry, comparing H-score in tumor (*n* = 564, including primary and metastatic lesions) and non-tumor (*n* = 16) tissues. The tumor samples comprised primary tumor specimens from 318 patients, with multiple samples from a subset of patients, as well as metastatic samples. The non-tumor samples comprised matched, uninvolved colon tissues from 16 of the 318 patients. While we had previously observed PSB7 overexpression in both the cytoplasm and nuclei of colorectal cancer samples, the more striking expression was in the cytoplasm (Figures 1D–G). H-score for PSB7 levels was clearly heterogeneous among the different colorectal cancer tumor samples, with a large range, and the level was significantly higher in tumor samples, especially in metastatic lesions (Figure 1A). Among the 41 samples where we had both the primary and the matched metastatic lesion, the average difference in H-scores between the matched metastatic lesion and the primary lesion was −4; the H-score was higher in the metastatic lesion in 15 of the 41 cases, and the average difference for the 15 cases was 56. These differences between primary and metastatic lesions were not statistically significant by Wilcoxon signed rank test (*p* = 0.6532, Figure 1B).

We then focused on the PSB7 H-scores in primary tumor samples (*n* = 318) and examined the relationship between the PSB7 H-scores with different patient and disease characteristics. PSB7 H-scores were higher with high AJCC-stage disease, but this difference was only observable between stage IV and lower stages (Figure 1C, Table 1). There was no significant difference in PSB7 H-scores with regards to the tumor T-stage, tumor grade, tumor size, tumor site (left vs. right colon), or microsatellite status (MSI vs. MSS) (Table 1). There were also no significant differences in PSB7 H-scores with regards to the patient sex or age (dichotomizing based on cut-off ages of 60, 65, and 70 years at diagnosis). There was also no significance difference among patients who received chemotherapy vs. those who did not receive chemotherapy.

**Table 1:** Comparison of patient and disease characteristics among the patients with high and low PSB7 H-score values.

		**>Median**	**≤Median**	***p* value**
Stage at diagnosis	I II III IV	10 (11.1%) 14 (15.6%) 32 (35.6%) 34 (37.8%)	34 (14.9%) 61 (26.8%) 97 (42.5%) 36 (15.8%)	0.0004
Pathologic T (tumor) stage	1 2 3 4	2 (2.4%) 11 (12.9%) 48 (56.5%) 24 (28.2%)	14 (6.7%) 34 (16.3%) 118 (56.5%) 43 (20.6%)	0.2208
Grade	1 2 3	4 (4.6%) 79 (90.8%) 4 (4.6%)	17 (8.1%) 170 (81%) 23 (11%)	0.0832
Size (cm)	Mean ± SEM	4.67 ± 0.1	4.59 ± 0.2	0.9675
Age (years)	Mean ± SEM	67.0 ± 1.3	66.6 ± 0.8	0.9681
Gender	Female Male	44 (48.9%) 46 (51.1%)	109 (47.8%) 119 (52.2%)	0.9012
Location	Left Right	39 (43.3%) 51 (56.7%)	108 (47.4%) 120 (52.6%)	0.5151
Adjuvant chemotherapy	Yes No Not known	41 (45.6%) 44 (48.9%) 5 (5.6%)	87 (38.2%) 138 (60.5%) 3 (1.3%)	0.1096
Microsatellite status	MSI MSS Not known	7 (7.8%) 73 (81.1%) 10 (11.1)	26 (11.4%) 197 (86.4%) 5 (2.2%)	0.5373
Death	Censored Death	33 (36.7%) 57 (63.3%)	114 (50%) 114 (50%)	0.0308

### Prognostic Significance of PSB7 in Colorectal Cancer Patients

**Figure 2:**
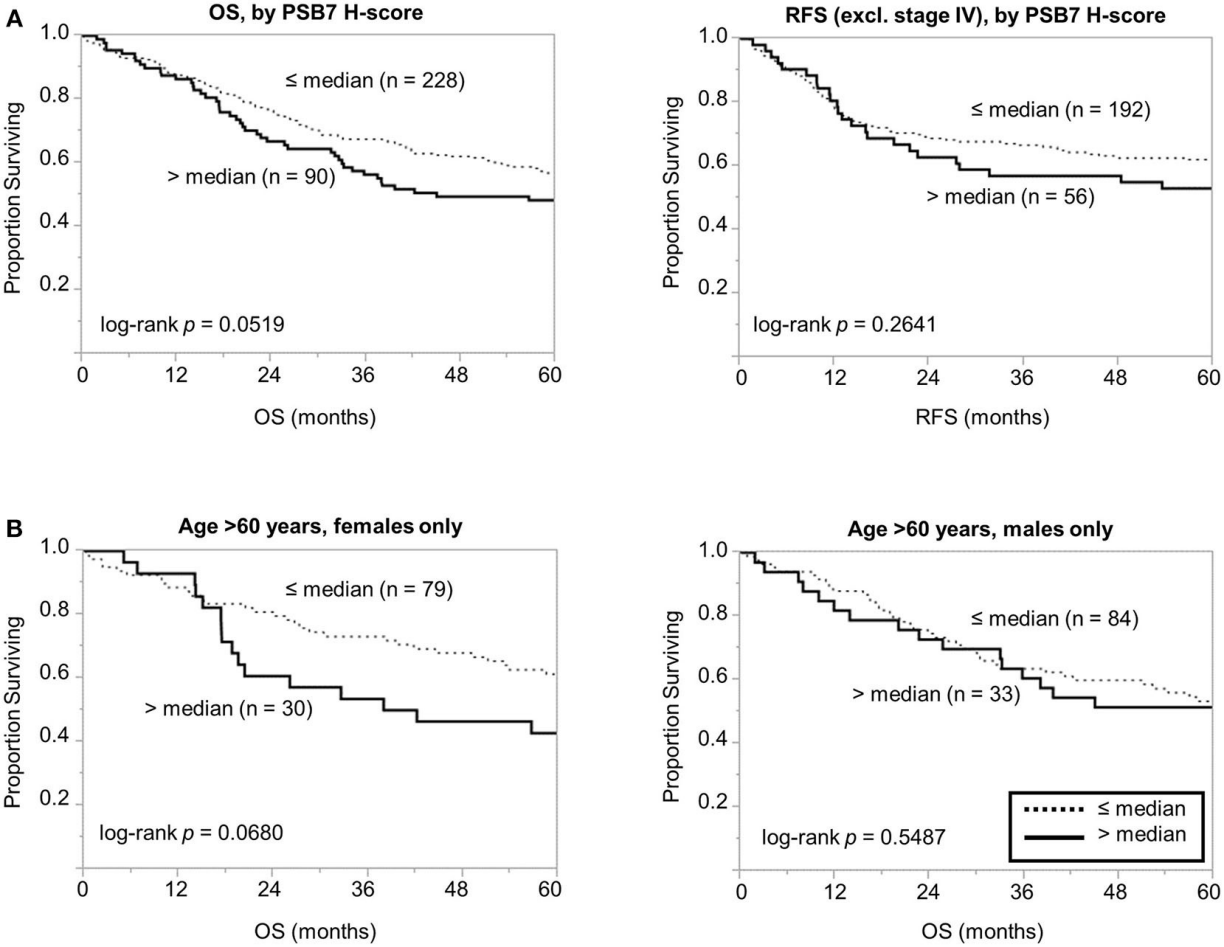
Comparison of overall and recurrence-free survival among colorectal cancer patients dichotomized based on PSB7 H-scores. **(A)** Comparison of overall survival (OS) and recurrence-free survival (RFS) between patients with high (solid line) and low (dotted line) PSB7 levels. **(B)** Comparison of overall survival among older (aged >60 years at diagnosis) female and male patients with high (solid line) and low (dotted line) PSB7 levels.

We then focused on the PSB7 H-scores in primary tumor samples (*n* = 318) and examined the prognostic value of PSB7. Patients were categorized into High vs. Low PSB7 groups, based on the PSB7 H-scores of the primary lesions with respect to the median PSB7 H-score. High PSB7 levels were associated with a greater number of deaths during our follow-up period of up to 10 years (Table 2). High PSB7 was associated with worse overall survival (OS) by univariate analysis (Table 2), but the difference was not significant by log-rank analysis (*p* = 0.0519, Figure 2A), and there was no significant difference in the recurrence-free survival (RFS, *p* = 0.2641) (Figure 2A). Interestingly, when we performed sub-group analyses, the difference in OS was more visually apparent when we focused on female patients, while the OS difference disappeared when we examined male patients only (data not shown). Furthermore, among the female patients, the OS difference was only apparent among the older (age >60 years at diagnosis) female patients (Figure 2B), and such a trend was not observed younger (age ≤ 60 years at diagnosis) female patients or male patients. However, the difference in OS in older female patients remained non-significant (*p* = 0.0680), presumably related to the smaller number of patients.

**Table 2:** Univariate and multivariate analysis of overall survival of older (>60 years of age at diagnosis) female patients who received adjuvant chemotherapy (*n* = 66)^*^.

	OS^**^	
	**Univariate HR**	**Multivariate HR**
PSB7 high	3.34 (1.17–9.18) *p* = 0.0263	3.75 (0.95–14.6) *p* = 0.0579
Clinical stage IV	13.00 (4.09–42.77) *p* < 0.0001	25.84 (5.13–158.92) *p* < 0.0001
Histologic grade >2	1.29 (0.20–4.68) *p* = 0.7477	2.32 (0.32–10.68) *p* = 0.3547
Size >5 cm	0.66 (0.15–2.07) *p* = 0.4991	0.43 (0.08–1.82) *p* = 0.2631
Right colon	1.09 (0.56–2.21) *p* = 0.8049	3.81 (0.82–20.89) *p* = 0.0887

* *Cox proportional hazards method. HR, hazard ratio*.

** *Overall survival of female patients aged >60 years*.

**Figure 3:**
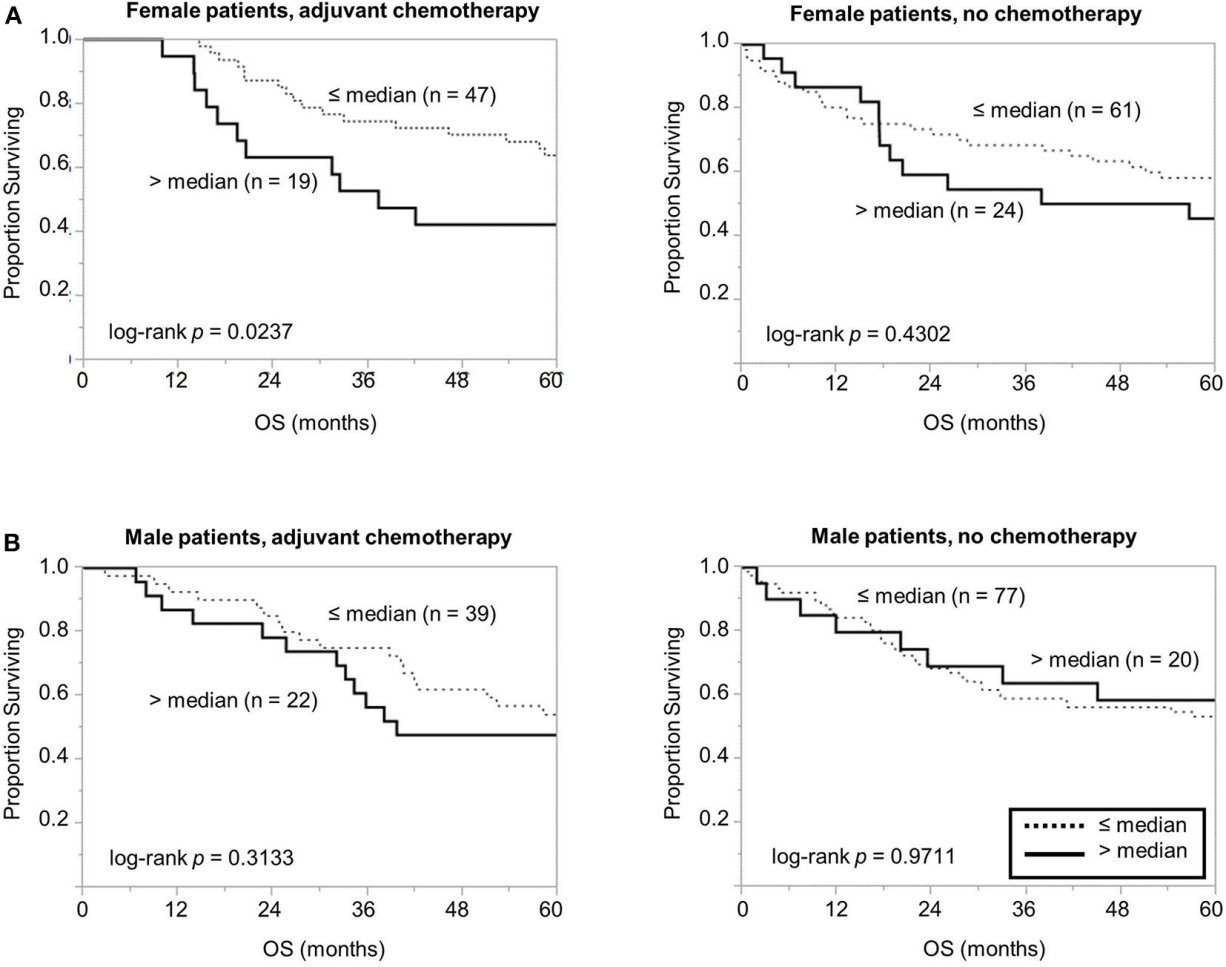
Comparison of overall survival based on PSB7 levels, examining specific groups of patients based on whether received adjuvant chemotherapy. **(A)** Comparison of overall survival in female patients who received chemotherapy with high (solid) and low (dotted) PSB7 levels and comparison in female patients that did not receive adjuvant chemotherapy. **(B)** Comparison in male patients that received adjuvant chemotherapy and comparison in male patients that did not receive adjuvant chemotherapy.

Considering the association between high PSB7 and chemotherapy resistance in breast cancer models (4), we next examined the prognostic significance of PSB7 in patients who received chemotherapy. Interestingly, among the female patients who received chemotherapy, high PSB7 was highly predictive of poor OS, while it had no significant prognostic power among female patients who did not receive chemotherapy or male patients (Figure 3). When we focused on older female patients who received chemotherapy, high PSB7 was associated with worse OS by univariate analysis, but it did not retain its significance on multi-variate analysis (*p* = 0.0579) in a model that included TNM stage, tumor grade, tumor size and tumor location.

**Figure 4:**
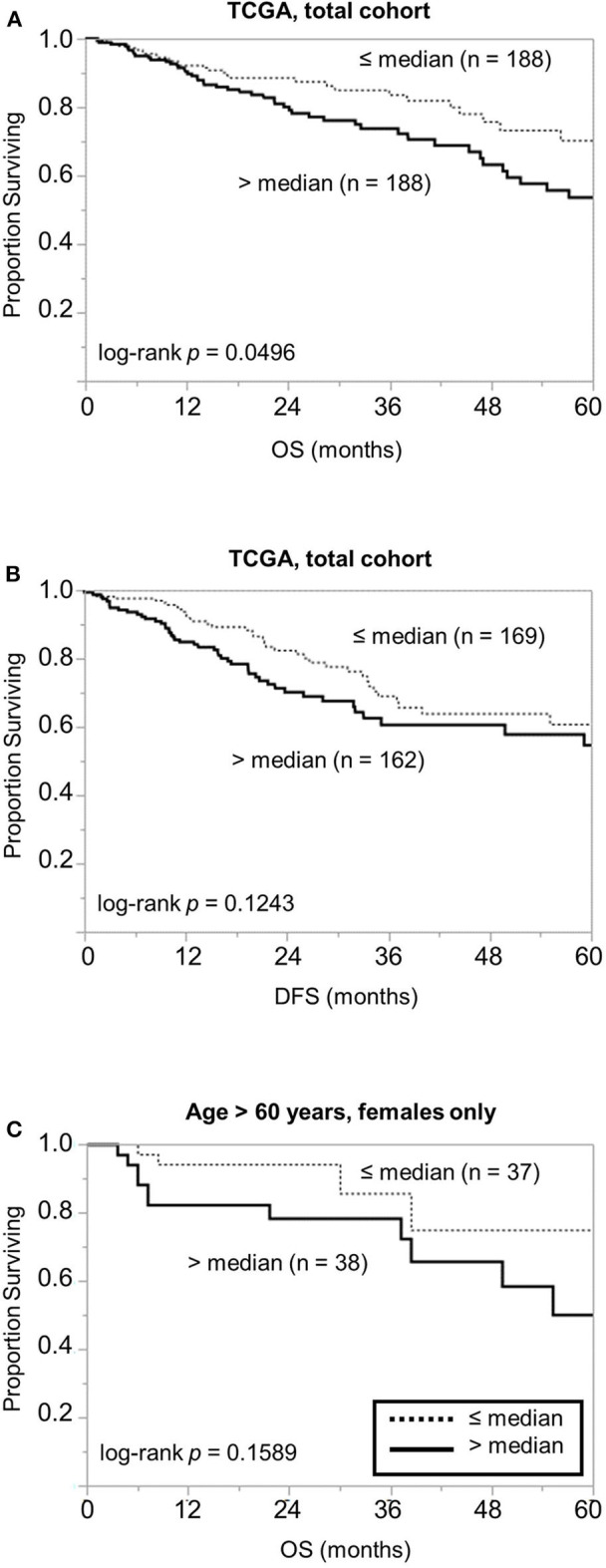
Examination of the GSE17538 data for the prognostic significance of the *PSMB7* gene expression levels. Comparison of **(A)** overall survival (OS) and **(B)** disease free survival (DFS) in all patients with high (solid) and low (dotted) *PSMB7* expression in the GSE17538 cohort with respect to overall stage. **(C)** Comparison of OS in the subgroup of females aged >60 years at diagnosis with high (solid) and low (dotted) *PSMB7* expression.

To examine the above findings in another cohort, we examined the prognostic significance of *PSMB7* mRNA levels in The Cancer Genome Atlas (TCGA) cohort. In a cohort of 376 patients, high *PSMB7* level was associated with worse OS; while the difference was relatively modest, it was statistically significant (*p* = 0.0496) (Figure 4A). The distinction was not significant for disease-free survival (DFS), similar to our findings with the protein levels (Figure 4B). When we performed sub-group analyses, focusing on female and older patients, the statistical significance was lost, despite the larger difference in median time (41.3 months for high *PSMB7* mRNA vs. not reached for the low *PSMB7* mRNA group, Figure 4C).

## Discussion

The ubiquitin-proteasome system influences the colorectal epithelium in a number of ways, and it is thought to contribute to its carcinogenesis by regulating the Wnt/β-catenin/APC signaling and the NF-κB pathways, which pathways that regulate proliferation and differentiation of the colonic epithelium (8). Proteasome inhibitors, either as monotherapy or in combination with other agents, have been found to be toxic to colorectal cancer cells, with a number of mechanisms implicated in the mechanism, including suppression of the NF-κB signaling pathway, proteasome-dependent caspase-3 degradation, as well as stabilization of pro-apoptotic Bcl-2 family proteins (9–12). Depletion of PSMA7 using shRNA was also found to inhibit colorectal cancer cell growth *in vitro* (13).

Study of proteasome inhibitors in CRC appears to be largely limited to pre-clinical, and generally in combination with other agents. It appears that proteasome inhibitors are largely ineffective in CRC; for example, phase II study of bortezomib with/without irinotecan had little activity in relapsed/refractory colorectal cancer (14). While the mechanism of clinical resistance to proteasome inhibitors is unknown; one potential mechanism may be related to the stabilization of the anti-apoptotic protein Mcl-1 (15).

As a prognostic marker, however, we describe PSB7 to be a prognostic marker in a specific subset of patients, namely female patients who are either older or who received chemotherapy. Despite the difference in prognostic value of PSB7 with respect to the patient sex, there were no differences observed with respect to the PSB7 H-scores with regards to the age or sex, suggesting that it is the effects of high PSB7, rather than mechanisms driving the high PSB7 levels, that contribute to the poor survival in colorectal cancer patients. Examining the PSB7 levels with respect to various patient and disease characteristics, high PSB7 were seen with higher stage disease, but this trend was only significant in stage IV disease, suggesting that the upregulation of PSB7 is a late phenomenon in the disease progression. While PSB7 levels were higher in metastatic lesions compared to primary tumors when examined *en bloc*, PSB7 levels were comparable between the metastatic lesions and the matched primary tumors. This suggests that PSB7 upregulation may be contributing to hematogenous metastasis in colorectal cancer.

Based on the correlation between high stage disease and high PSB7 levels, the association between high PSB7 and poor OS is not surprising, but we were surprised by the sex difference in the prognostic value of PSB7. While the prognostic value of high PSB7 was not particularly robust in general, its significance was markedly stronger among older patients and among patients who received chemotherapy. Overexpression of a protein can result in various phenotypes in a number of ways, and overexpression can result in both loss and gain of functions (16, 17). One mechanism involves the loss of a protein complex function by unbalanced overexpression of a single subunit, such as that observed with unbalanced histone overexpression (17). PSB7 is a component of the immunoproteasome, as well as the constitutive 26S proteasome, and overexpression of PSB7 may result in loss of function in either complexes. There are multiple mechanisms by which cancer cells escape immune surveillance, and suppression of the immunoproteasome appear to be one of the mechanisms. For example, in acute promyelocytic leukemia (APL), PML/RARα appears to directly control the transcription of the immunoproteasome subunits, contributing to lowered expression of PSMB8-10 in APL cells (18). Disruption of the immunoproteasome by constitutive expression of one of the proteasome subunits may be a possible mechanism by which colorectal cancer cells may be able to escape immune surveillance, especially when coupled with other aging-related changes in the immune system, including reduced production of lymphocytes and dysregulated signaling in T-cells and polymorphonuclear neutrophils (19, 20). Genetic polymorphism in *PSMB8* (LMP7, PSMB5i, β5i) has also been described in colorectal cancer patients associated with reduced inducibility in response to IFN-γ, another potential mechanism by which colorectal cancer cells may be able to escape immune surveillance (21). On the other hand, PSMB9 (LMP2, PSMB6i, β1i) was found to be frequently expressed in colorectal cancers, although no clear clinico-pathological correlations were apparent with its levels (22). It has also been hypothesized that the immune response plays a role in chemotherapy response through a number of different mechanisms, and chemotherapy-induced immunogenic cell death has been described in response to at least some of the chemotherapeutic agents, including oxaliplatin and 5-fluorouracil (23). It thus may be possible that the disruption of the immunoproteasome may be a mechanism by which cancer cells can dampen the anti-neoplastic response of the immune response while receiving chemotherapy.

In summary, we report the prognostic significance of PSB7 protein expression, which was more marked among older female patients, especially those treated with adjuvant chemotherapy. This is an interesting observation, given the known relationship between patient sex, the immune system, and the role of PSB7 in antigen presentation. Further studies will be needed to examine whether PSB7 quantification in prospective patient cohorts will have prognostic or predictive value for therapeutic decision-making. In this regard, patients receiving immune checkpoint inhibitors would be of high interest.

## Data Availability Statement

All datasets generated for this study are included in the article/supplementary material.

## Ethics Statement

This study was approved by UHN's Research Ethics Board (REB), and all experiments were carried out according to relevant institutional regulations and guidelines. The study used only fully de-identified retrospective material that had been collected as part of routine clinical care. Thus, the UHN REB had determined that informed consent was waived.

## Author Contributions

J-YY carried out the experiments, performed data analysis, and wrote a draft of the paper. JW advised on the project and edited the paper. MR supervised the project, analyzed the data, and wrote the final manuscript. All authors contributed to the article and approved the submitted version.

## Conflict of Interest

JW is founder of and equity holder in Curandis. MR is member of the Scientific Advisory Boards of Proscia and Trans-Hit. None of these companies had any influence in support, design, execution, data analysis, or any other aspect of this study. The remaining author declares that the research was conducted in the absence of any commercial or financial relationships that could be construed as a potential conflict of interest.
